# Next-Generation Sequencing-Aided Rapid Molecular Diagnosis of Occult Macular Dystrophy in a Chinese Family

**DOI:** 10.3389/fgene.2017.00107

**Published:** 2017-08-25

**Authors:** Yu-He Qi, Feng-Juan Gao, Fang-Yuan Hu, Sheng-Hai Zhang, Jun-Yi Chen, Wan-Jing Huang, Guo-Hong Tian, Min Wang, De-Kang Gan, Ji-Hong Wu, Ge-Zhi Xu

**Affiliations:** ^1^Eye Institute, Eye and ENT Hospital, College of Medicine, Fudan University Shanghai, China; ^2^Shanghai Key Laboratory of Visual Impairment and Restoration, Science and Technology Commission of Shanghai Municipality Shanghai, China; ^3^Key Laboratory of Myopia, Ministry of Health Shanghai, China

**Keywords:** occult macular dystrophy, gene mutations, molecular diagnosis, *ABCA4*, *RP1L1*

## Abstract

**Purpose:** To show early, rapid and accurate molecular diagnosis of occult macular dystrophy (OMD) in a four-generation Chinese family with inherited macular dystrophy.

**Methods:** In the current study, we comprehensively screened 130 genes involved in common inherited non-syndromic eye diseases with next-generation sequencing-based target capture sequencing of the proband of a four-generation Chinese family that has suffered from maculopathy without a definitive diagnosis for over 10 years. Variants were filtered and analyzed to identify possible disease-causing variants before validation by Sanger sequencing.

**Results:** Two heterozygous mutations—*RP1L1* c.133 C > T (p.Arg45Trp), which is a hot spot for OMD, and *ABCA4* c.6119 G > A (p.Arg2040Gln), which was identified in Stargardt’s disease were found in three patients, but neither of the mutations was found in the unaffected individuals in the same family, who are phenotypically normal or in the normal control volunteers.

**Conclusion:** These results cannot only confirm the diagnosis of OMD in the proband, but also provide presymptomatic diagnosis of the proband’s children before the onset of visual acuity impairment and guidance regarding the prognosis and management of these patients. Heterozygous mutations of *RP1L1* c.133 C > T (p.Arg45Trp) and *ABCA4* c.6119 G > A (p.Arg2040Gln) are likely responsible for OMD. Our results further extend our current understanding of the genetic basis of OMD, and emphasize the importance of molecular diagnosis and genetic counseling for OMD.

## Introduction

Occult macular dystrophy (OMD) is an unusual, inherited or sporadic macular dystrophy characterized by an almost normal-appearing fundus and fluorescein angiography, with aggressive decreases in binocular visual acuity (BCVA) (Miyake et al., 1996). The age of onset varied from 6 to 81 years old (Akahori et al., 2010). OMD patients show normal full-field electroretinograms (ERGs), but a markedly decline in macular responses in multifocal ERG (Tsunoda et al., 2012; Nakanishi et al., 2015). Photoreceptor and outer nuclear layer (ONL) abnormalities are the main pathologic changes in OMD; thus spectral domain optical coherence tomography (SD-OCT) and mfERG are considered the best assessments for clinical diagnoses (Park et al., 2010; Ahn et al., 2013; Sisk and Leng, 2014; Nakanishi et al., 2015). However, these diagnostic tools are time consuming and require both equipment and resources. In addition, patients with poor fixation due to low vision or patients who are too young to cooperate with visual testing often show variable results on mfERG. Moreover, due to the clinical heterogeneity of retinal diseases, fast and accurate diagnosis and identification are often difficult. Most importantly, early-stage OMD, with no visual impairment exhibits phenotypic overlap with numerous retinal diseases, such as cone-rod dystrophy, early changes in age-related macular degeneration and Stargardt’s disease (SD) (Pang and Lam, 2002), and is not easily detected. Therefore, genetic diagnosis is an essential first step for the early diagnosis and confirmation of OMD. Genetic testing is extremely curial for genetic counseling, risk assessment, individualized therapy and carrier screening of OMD patients and their family members. With the advent of precision medicine and gene diagnosis technology, a thorough description of any patient’s disease should include both genetic and clinical information (Ashley, 2015; Barlas, 2015; Collins and Varmus, 2015; Ghitza, 2015).

The hereditary mode of OMD is autosomal dominant in numerous patients and sporadic in others (Akahori et al., 2010; Davidson et al., 2013). The RP1-like protein 1 (*RP1L1*) gene (OMIM 608581) is the gene most commonly associated with OMD (Davidson et al., 2013), and many mutations in *RP1L1* have been reported, such as p.Arg45Trp (Akahori et al., 2010; Hayashi et al., 2012; Tsunoda et al., 2012; Ahn et al., 2013; Okuno et al., 2013), p.S1199P (Takahashi et al., 2014), p.S1199C (Kabuto et al., 2012), p.W960R (Akahori et al., 2010), p.Q2311P (Ahn et al., 2013), and p.S676C (Ahn et al., 2013). The most frequent mutation is p.Arg45Trp in exon 2. However, p.Arg45Trp is involved in only approximately 37% of Korean, white European and British OMD cases (Akahori et al., 2010; Ahn et al., 2013; Davidson et al., 2013). Davidson et al. (2013) suggested that the Arg45Trp mutation is a risk factor, not a causative mutation, for OMD. Therefore, OMD is considered to be a genetically heterogeneous disease.

In view of the substantial clinical and genetic complexity underlying OMD, molecular diagnosis is an effective supplementary diagnosis method. In this study, we performed molecular diagnosis of a four-generation Chinese family by next-generation sequencing (NGS)-based target captures sequencing and a complete ophthalmological examination. Two heterozygous mutations—*RP1L1* c.133 C > T (p.Arg45Trp) and ATP-binding cassette transporter 4 (*ABCA4*) c.6119G > A (p.Arg2040Gln) were identified in three patients, including individuals with normal vision. These results cannot only confirm and provide presymptomatic diagnosis and detection of OMD but also expand our knowledge of genotype–phenotype correlations and emphasizes the significance of molecular diagnosis and genetic counseling for OMD.

## Materials and Methods

### Subjects and Ethics Statement

The current study was performed in accordance with the Code of Ethics of the World Medical Association (Declaration of Helsinki) for medical research involving human subjects and was approved by the Ethics Committee of the Eye and ENT Hospital of Fudan University. Written informed consent was obtained from all participants or their guardians.

### Clinical Evaluations and Sample Collection

Our study involved seven family members from four generation, who underwent detailed history ophthalmic examination, including BCVA testing, color vision (Ishihara color plate), slit lamp biomicroscopy, tonometry (Humphrey VisualField Analyzer, Carl Zeiss, Inc., Dublin, CA, United States), dilated fundus examination, SD-OCT (Spectralis HRA + OCT, Heidelberg, Engineering, Inc., Heidelberg, Germany), OCT angiography (OCTA), visual field tests with RTVue XR Avanti OCT with AngioVue (Optovue, Fremont, CA, United States), fundus autofluorescence (FAF, Spectralis HRA +OCT; Heidelberg, Germany), full-field ERG and multifocal ERG (mfERG, LKC UTAS E3000 LKC Technologies, Inc., United States). We also examined 256 normal control volunteers from China, with a normal phenotype in visual acuity testing and visual field measurement and no other obvious serious diseases. Family and medical history, including subjective degree of vision loss, age of onset and other related clinical manifestations, was obtained. Blood samples were collected from the peripheral blood and stored at 4°C before further analysis.

### Genetic Analyses

Genomic DNA from the family was extracted from the peripheral blood on April 25, 2016 using a standard method (Ma et al., 2015). The proband was analyzed first; we designed a high-throughput microarray with exon-capture and NGS targeting of the 130 genes that are most frequently involved in common inherited non-syndromic eye diseases (the capture probes were custom designed and produced by BGI, **Supplementary Table S2**). Then, library preparation, qualification, and NGS of the targeted sequences were further conducted on an Illumina Hiseq 2000 platform (Illumina, Inc., San Diego, CA, United States) in collaboration with BGI-Shenzhen (Shenzhen, Guangdong, China) as detailed previously (Chen et al., 2013, 2015). The following 5 databases were then used for annotation of all identified variants with minor allele frequency (MAF) > 0.1% to eliminate benign variants: dbSNP137^1^, HapMap Project^2^, 1000 Genomes Project^3^, YH database^4^, and Exome Variant Server^5^. Finally, the variant prioritizations were performed, combining total depth, quality score, MAF, potential deleterious effect and the existence of mutation reports in common databases such as the Human Gene Mutation Database (HGMD), Retinal Information Network (RetNet), ClinVar or Online Mendelian Inheritance in Man (OMIM). For variants that passed the initial filtration, we used Sanger sequencing for verification of variants within other family members and 256 normal control volunteers. Sanger sequencing was conducted as previously described (Zhao et al., 2006). Evolutionary conservation of the mutations was performed online^6^.

## Results

### Clinical Examination and Pedigree Analysis

**FIGURE 1:**
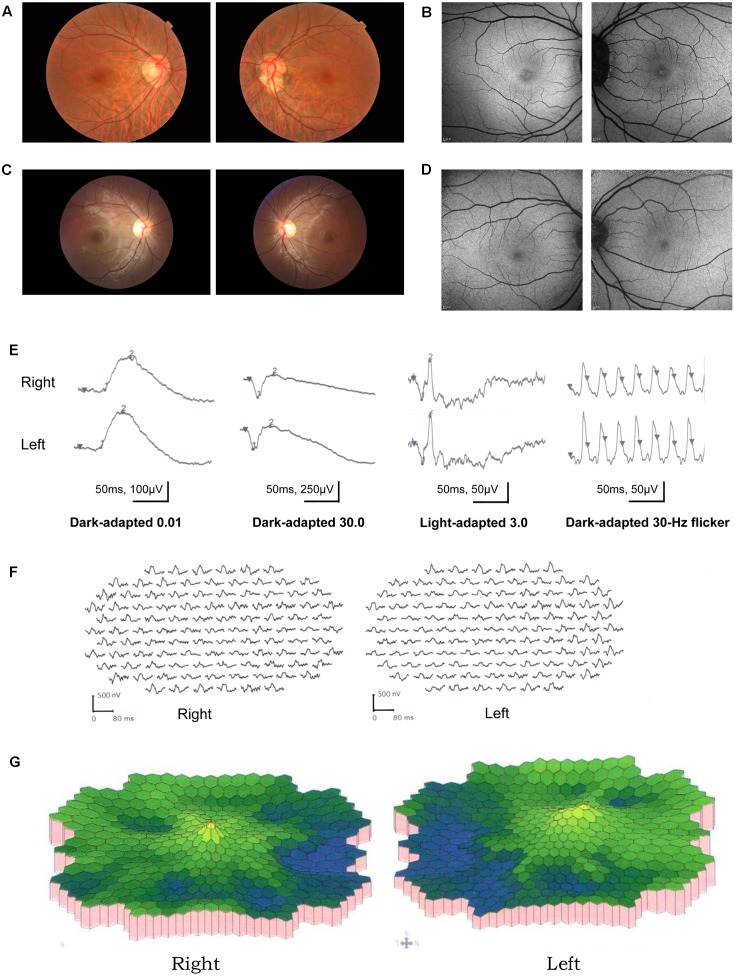
Results of ocular examination of the proband (P5) and his son (P7). Photographs of the fundus **(A,C)** and fundus autofluorescence **(C,D)** of both eyes of the P5 **(A,B)** and P7 **(C,D)**. **(E)** (P5) Full-field rod, mixed rod-cone, and cone ERGs, and 30-Hz flicker responses. All the responses were normal in both eyes. **(F,G)** (P5). Amplitudes in the rings 1–3 of both eyes were significantly reduced, with the right eye more seriously affected.

**FIGURE 2:**
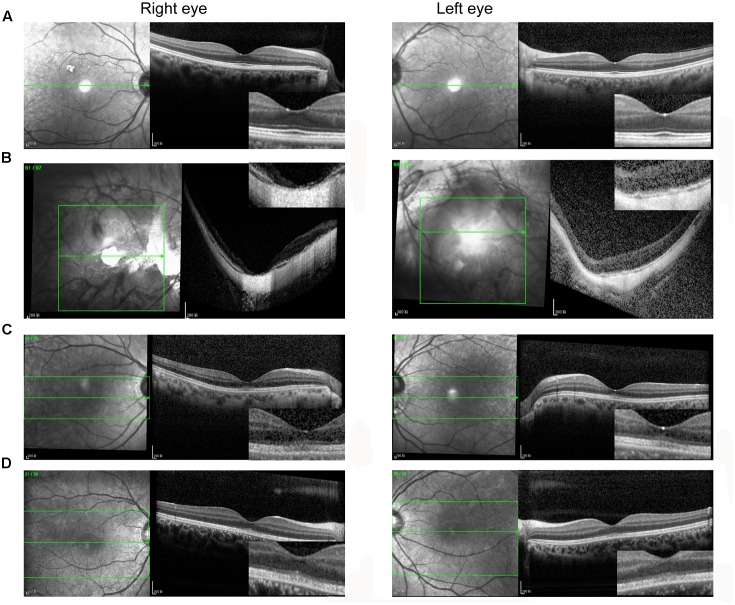
Spectral domain optical coherence tomography images of the fovea. **(A)** P3; **(B)** P4; **(C)** P5; **(D)** P7. **(A,B)** The retinal thickness and structures in both the fovea and the perimacular region were normal **(A)**, but severely damaged in **(B)**. **(C,D)** The interdigitation and ellipsoid zones were disrupted at the fovea but were still visible in the perimacular region.

**FIGURE 3:**
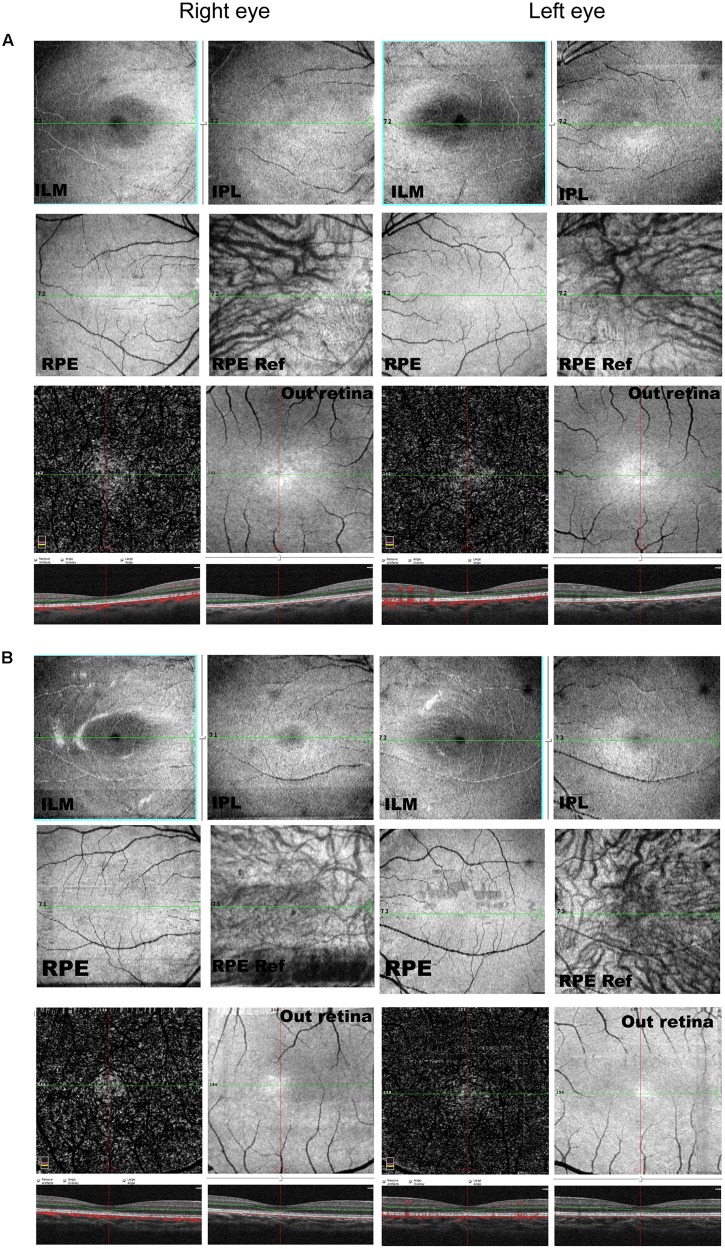
OCTA of P5 **(A)** and P7 **(B)**. Data were set to flatten the inner limiting membrane (ILM), inner plexiform layer (IPL), and retinal pigment epithelium (RPE) from the inner to outer retina, and no aberrant vascular networks were detected. ILM, internal limiting membrane; IPL, inner plexiform layer.

A total of 256 normal control volunteers and seven members from a four-generation family were included in this study. The clinical characteristics of the four affected family members are listed in **Table 1**. The proband (P5), a 38-year-old man, has suffered from a progressive decrease in visual acuity in both eyes over 10 years without a definite diagnosis. Ophthalmic examinations revealed a BCVA of 0.5 (OD:1.50 DS; OS:0.25 DS) in both eyes. Intraocular pressure (IOP) was 11 mm Hg in the right eye and 12 mm Hg in the left eye. The proband’s anterior segments and media examination were unremarkable. His fundus (**Figure 1A**) and visual field (data not shown) revealed no abnormalities, while FAF demonstrated a faint hyperfluorescent ring resembling a bull’s eye (**Figure 1B**). Scotopic and photopic full-field ERGs showed normal responses (**Figure 1E**), while mfERG showed that the amplitudes in the rings 1–3 of both eyes were significantly reduced, with the right eye more seriously affected (**Figures 1F,G**), indicating dysfunction of the central retina (Fujii et al., 1999; Piao et al., 2000). SD-OCT revealed disruption of the interdigitation and ellipsoid zones at the fovea, but both were still visible in the perimacular region (**Figure 2C**). For further observation of retinal perfusion in the macular region, OCTA was performed on P5 and P7 (**Figures 3A,B**), the data were set to flatten the inner limiting membrane (ILM), inner plexiform layer (IPL), and retinal pigment epithelium (RPE) from the inner to outer retina, and no aberrant vascular networks were detected. The fundus (**Figure 1C**), FAF (**Figure 1D**), SD-OCT (**Figure 2D**) and OCTA (**Figure 3B**) results from P7, a 9-year-old patient, whose BCVA was 1.0 in both eyes, were similar to those from P5. Eight months later, the BCVA of P7 began to decrease (0.8 in both eyes). The proband’s father (P3), who was 62 years old, had a BCVA of 0.6 in the right eye and 0.8 in the left. SD-OCT showed that the retinal thickness and structures in both the fovea and the perimacular region were normal (**Figure 2A**), but severely damaged in P4 (**Figure 2B**), whose BCVA was 0.08 in both eyes. P4 is the proband’s mother, 63-year -old, she suffered from vision problems when she was 30 years old. The detailed clinical characteristics of P3, P4, P5, P7 are listed in **Table 1**.

**Table 1:** Clinical characteristic of patients.

Patients	P3	P4	P5	P7
Age (years)	62	63	38	9
Onset (years)	–	30	10	–
BCVA (OD/OS)	0.6/0.8	0.08/0.08	0.5/0.5	1.0/1.0
Visual field	Normal	–	Normal	Normal
Fundus	Normal	Macular atrophy	Normal	Normal
OCT	Normal	Retinal thickness and structures were severely damaged	Interdigitation and ellipsoid zone were disrupted	Interdigitation and ellipsoid zone were disrupted
FAF	–	–	Abnormal	Abnormal
OCTA	–	–	Normal	Normal


*OCT, optic coherence tomography; FAF, fundus autofluorescence; OCTA, optical coherence tomography angiography. – we did not get.*

### Genetic Analyses

**FIGURE 4:**
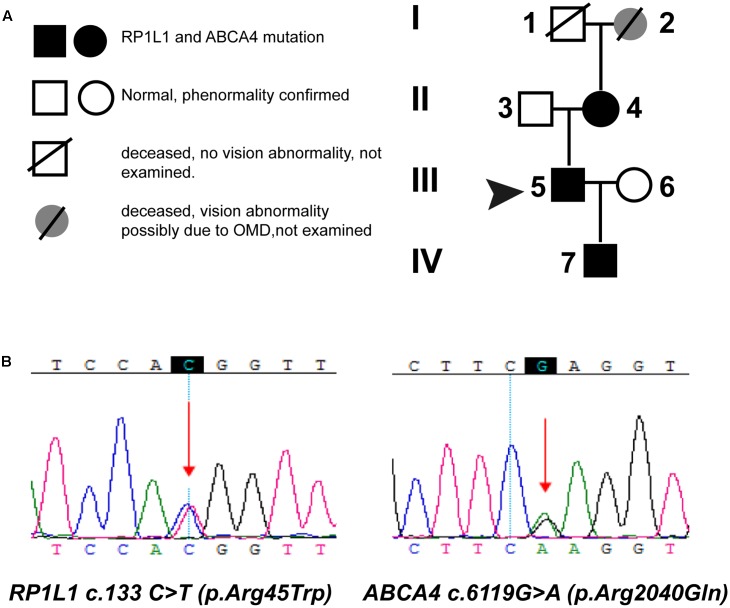
Pedigrees **(A)** and identified mutations **(B)**. **(A)** Circles represent females, and squares represent males. Filled symbols represent affected patients, and empty symbols represent normal controls. **(B)** Sequencing results of the mutations in the *ABCA4* and *RP1L1* gene. Arrows indicate the position of the mutated nucleotide.

**FIGURE 5:**
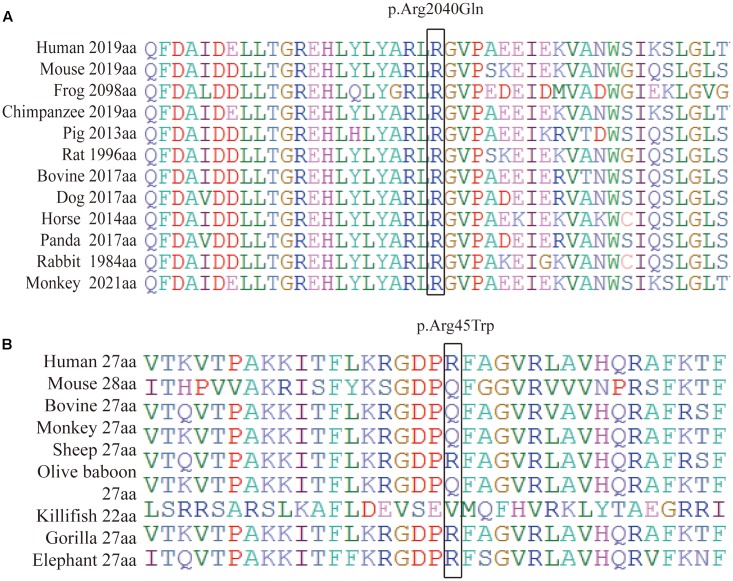
Protein sequence alignment of human ABCA4 **(A)** and RP1L1 **(B)** with their orthologs. **(A)** Shows that codon 2040 (isoleucine) of ABCA4 and its subsequent sequences were highly conserved amino acids across different species. **(B)** Reveals that mutations of c.133 C > T in the RP1L1 gene were not conserved in the nine species studied.

We performed a targeted NGS approach on the proband (P5). The targeted gene length was 514,593 bp and the mean depth of target region was 187.67 X, with 99.61% coverage. After the data acquisition and analysis, an average of 169 raw variants were initially obtained (**Supplementary Table S1**). Finally, by further bioinformatics analysis, two missense mutation c.133 C > T in the *RP1L1* gene (Akahori et al., 2010; Ahn et al., 2013; Davidson et al., 2013) and c.6119 G > A in the *ABCA4* gene (Xu et al., 2014; Zernant et al., 2014; Jiang et al., 2016) were identified as potentially pathogenic mutations (**Table 2** and **Figure 4B**). These mutations, c.133 C > T and c.6119 G > A, were extremely rare in the control population, with a frequency of 0.0009 and 0 in 1000 Genomes Project, respectively. Moreover, the mean occurrence frequency of the two mutations in the SNV database of over 200 Chinese individuals was less than 0.01. Further analysis using PolyPhen-2 and SIFT predicted that both mutations were pathogenic. Sanger sequencing was then performed to validate the variant in the studied family and the 256 normal control individuals. The proband’s mother (P4) and his son (P7) carried the two mutations in the heterozygous state, but the mutations were not found in his father or the 256 unrelated healthy individuals, who were phenotypically normal (P3, **Figure 4A**). Multiple orthologous sequence alignment (MSA) revealed that codon 2040 of *ABCA4* and its subsequent sequences were highly conserved amino acids across different species (**Figure 5A**), suggesting that mutations of those codons may lead to a deleterious effect. The *RP1L1* c.133 C > T mutation was not conserved in the nine species analyzed in this study (**Figure 5B**).

**Table 2:** Mutations identified in the present study.

Gene	NCBI reference sequence	Nucleotide change	Amino acid substitution	Chromosomal location	Gene subregion	Allele status
*ABCA4*	NM_000350	c.6119 G > A	p.Arg2040Gln	Chr1:94471025	EX44/CDS44	Het
*RP1L1*	NM_178857	c.133 C > T	p.Arg45Trp	Chr8:10480579	EX2/CDS1	Het


*Het, heterozygous.*

## Discussion

Next-generation sequencing, which greatly intensely enhanced sequencing output with lower costs, is a high-throughput approach capable of efficiently sequencing large gene pools. NGS has enabled the identification of many rare disease genes (Roosing et al., 2013). Therefore, NGS has become a powerful tool for elucidating comprehensive mutation profiles for heterogeneous diseases (Coppieters et al., 2014), including OMD. In this study, we comprehensively screened 130 genes involved in common, inherited, non-syndromic eye diseases and successfully identified 2 potentially causative mutations for OMD, c.133 C > T in the *RP1L1* gene and c.6119 G > A in the *ABCA4* gene. Based on the mutation analysis and the clinical assessment, we concluded that OMD is the exact diagnosis of the three members (P4, P5, P7) of the studied family.

The human *RP1L1* gene, spanning 50 kb on chromosome 8p, is encoded in four exons. *RP1L1* has the highest sequence similarity to retinitis pigmentosa 1 (RP1 OMIM ^∗^ 603937), with 48% identity and 61% similarity between the human and mouse sequences (Bowne et al., 2003; Conte et al., 2003). The length of *RP1L1* mRNA is over 7 kb, whereas the specific length is varies across individuals as a result of the presence of several length polymorphisms. The predicted molecular weight of the protein, encoded by *RP1L1* and with a minimal length of 2,400 amino acids, is predicted 252 kDa (Bowne et al., 2003). Immunohistochemistry shows that retina RP1L1 is present only in the retina, and localized to the connecting cilium of rod and cone photoreceptors, indicating a pathogenic function for the *RP1L1* mutation in OMD photoreceptor disturbances (Bowne et al., 2003; Akahori et al., 2010; Davidson et al., 2013; Takahashi et al., 2014). Amino acids 33–113 and 147–228, where the *RP1L1* p.Arg45Trp alteration resides, can interact with *RP1* to assemble and stabilize axonemal microtubules (Yamashita et al., 2009). Dominant mutations in *RP1L1* p.Arg45Trp were first reported in all affected individuals from three Japanese families with OMD (Akahori et al., 2010), but only four candidate genes were studied. Later, Seong Joon Ahn found that *RP1L1* p.Arg45Trp is involved in 36.8% of Korean OMD cases (Ahn et al., 2013), while different *RP1L1* mutations account for other cases. However, Ahn colleagues sequenced only the entire exons and flanking regions of the *RP1L1* gene in 19 Korean patients with OMD. Thus, the abovementioned studies cannot rule out the possibility of other mutations. Further genetic studies on more candidate genes with a larger population samples are needed. MSA revealed that *RP1L1* p.Arg45Trp is not conserved in the nine species analyzed in this study. This finding is consistent with previous studies showing that the *RP1L1* p.Arg45Trp variant may represent a risk factor for OMD rather than a causative mutation. Therefore, other causative genes may coexist.

The photoreceptor-specific *ABCA4* gene is localized at the rims of the outer segments of cone and rod photoreceptors, where it exclusively encodes a transmembrane protein exclusively (Papermaster et al., 1976; Cideciyan et al., 2009). As an active transporter of all trans-retinal (atRAL), *ABCA4* can flip *N*-retinylidene-phosphatidylethanolamine from the extracellular to cytoplasmic leaflet of internal disk membranes within photoreceptor outer segments (Tsybovsky et al., 2013). Mutations in *ABCA4* lead to the accumulation of toxic bisretinoid atRAL adducts of atRAL in photoreceptors and RPE (Cideciyan et al., 2015), and reduced BCVA reduced as the disease process is initiated near the center of the macula (Cideciyan et al., 2005; Huang et al., 2014). *ABCA4* mutations are one of the most frequent monogenetic pathogenesis for retinal degeneration (Cideciyan et al., 2009, 2012; Zangerl et al., 2010; Fujinami et al., 2013). The clinical manifestation of *ABCA4*-related retinopathy is variable, including autosomal recessive Stargardt’s disease (arSTGD), fundus flavimaculatus, autosomal recessive cone-rod dystrophy (arCRD) and autosomal RP (Jaakson et al., 2003; Valverde et al., 2007; Riveiro-Alvarez et al., 2013; Jiang et al., 2016). However, whether *ABCA4* also plays a role in OMD is unknown.

In this study, the proband has suffered from maculopathy without a definitive diagnosis for over 10 years, and his son was unaware of his visual abnormalities. Through mutation analysis, combined with clinical history and examination, we achieved an accurate molecular diagnosis of OMD in three family members carrying *RP1L1* p.Arg45Trp mutations in the family. Dominant mutations in the *RP1L1* gene were involved in100% of OMD cases in the current study and presumably arose for three reasons. First, genetic backgrounds or even non-genetic factors may affect the phenotypes of individuals with *RP1L1* variants, which has been proposed in previous studies. Consistent with this idea, significant phenotypic distinction has been detected in OMD patients, though with the same causative alleles in the same family (Tsunoda et al., 2012; Ahn et al., 2013). Moreover, in this study, the age of onset, disease progression and disease manifestations varied across the three patients. However, we cannot rule out the possibility that with increasing age and disease progression, visual performance may become similar across family members. Second, other causative genes may exist. Studies in Japanese have suggested that OMD is not a single disease caused by a specific gene mutation, such as *RP1L1* (Tsunoda et al., 2012). In the present study, we also found a heterozygous mutation c.6119 G > A in the *ABCA4* gene; therefore, we hypothesize that the *RP1L1* p.Arg45Trp or *ABCA4* p.Arg2040Gln mutations affects only part of the transcribed mRNA, and a certain amount of *RP1L1* or *ABCA4* protein is still sufficient to sustain proper cell function. When the two mutations are present simultaneously, abnormal protein may accumulates in cone and rod photoreceptors, or the two proteins or genes may interact, eventually leading to OMD. Our results are consistent with previous reports that the Arg45Trp mutation is a risk factor for OMD rather than a causative mutation (Piermarocchi et al., 2016). However, there may be other unknown mechanisms responsible for the observed phenotype, and further in-depth studies involving more family members and normal controls are needed.

## Conclusion

To the best of our knowledge, this study used the first NGS-based assay specifically designed for the confirmation and early diagnosis of OMD in a Chinese pedigree reported to date. In addition, we found that heterozygous mutations of *RP1L1* c.133 C > T (p.Arg45Trp) and *ABCA4* c.6119 G > A (p.Arg2040Gln) are likely responsible for OMD; this OMD genetic mutation pattern is novel, and the functions and interactions of *RP1L1* and *ABCA4* should be further investigated. This study not only provides a guide to the attending clinician on the management and prognosis of the patient, but also extends the phenotypic spectrum of *RP1L1*-associated OMD and enhances our current understanding of the genetic basis of OMD.

## Author Contributions

J-HW and G-ZX conceived and designed the experiments. F-YH and D-KG collected the clinical samples. J-HW, G-ZX, Y-HQ, and F-JG analyzed sequencing data. J-YC, G-HT, and MW recruited patients, performed clinical examination of patients and clinical interpretation. J-HW, Y-HQ, and F-JG drafted and revised the manuscript. All authors read and approved the manuscript.

## Conflict of Interest Statement

The authors declare that the research was conducted in the absence of any commercial or financial relationships that could be construed as a potential conflict of interest.

## Footnotes

1 http://hgdownload.cse.ucsc.edu/goldenPath/hg19/database/snp137.txt.gz

2 ftp://ftp.ncbi.nlm.nih.gov/hapmap

3 ftp://ftp.1000genomes.ebi.ac.uk/vol1/ftp

4 http://yh.genomics.org.cn/

5 http://evs.gs.washington.edu/EVS/

6 http://www.bioinformatics.org/strap/index2

## Supplementary Material

TABLE S1 The 169 mutations identified by NGS technology in the present study.

TABLE S2 The 130 eye disease-related genes included in the capture panel.
