# Supplementary material for: Next-Generation Sequencing-Aided Rapid Molecular Diagnosis of Occult Macular Dystrophy in a Chinese Family
Source: Front Genet. 2017 Aug 25;8:107. doi: 10.3389/fgene.2017.00107 (PMC5574873; doi:10.3389/fgene.2017.00107)
Supplement: TABLE S1 — The 169 mutations identified by NGS technology in the present study. [file Table_1.docx]

**1 Retinitis pigmentosa:**

RP1,RP2,RPGR,RHO,PRPH2,ROM1,RP9,IMPDH1,PRPF31,CRB1,PRPF8,TULP1,CA4,PRPF3,ABCA4,RPE65,EYS,CERKL,NRL,FAM161A,FSCN2,TOPORS,SNRNP200,SEMA4A,PRCD,NR2E3,MERTK,USH2A,PDE6B,PROM1,KLHL7,PDE6A,RGR,CNGB1,IDH3B,SAG,GUCA1B,CNGA1,BEST1,TTC8,C2orf71,ARL6,IMPG2,PDE6G,ZNF513,DHDDS,PRPF6,CLRN1,MAK,CDHR1,FLVCR1,RLBP1,SPATA7,AIPL1,LRAT,OFD1,CYP4V2

**2 Leber congenital amaurosis:**

AIPL1,CABP4,CEP290,CRB1,CRX,GUCY2D,IMPDH1,IQCB1,LCA5,LRAT,OTX2,RD3,RDH12,RPE65,RPGRIP1,SPATA7,TULP1,KCNJ13,NMNAT1

**3 Congenital stationary night blindness**

RHO,PDE6B,GNAT1,CACNA1F,NYX,GRM6,SLC24A1,TRPM1,CABP4,SAG,GRK1

**4 Achromatopsia**

CNGA3, CNGB3, GNAT2, PDE6H, PDE6C

**5 Cone -rod dystrophy**

PDE6H,KCNV2,GUCA1A,CACNA2D4,RPGR,CRX,ABCA4,CACNA1F,PDE6C,PITPNM3,GUCY2D,RIMS1,SEMA4A,RAX2,PROM1,RPGRIP1,CDHR1,RLBP1,UNC119,BEST1,CERKL,AIPL1

**6 Macular dystrophy**

ABCA4,FBLN5,RAX2,CNGB3,RPGR,BEST1,ELOVL4,PRPH2,RP1L1,CDH3,PROM1

**7 Stargardt disease**

ABCA4, PROM1, ELOVL4

**8 Retinal dystrophy**

RLBP1, OTX2, ABCA4, LRAT, EFEMP1, INPP5E

**9 Retinal ndegeneration**

NRL, C1QTNF5

**10 Fundus albipunctatus**

RDH5, RLBP1

**11 Fundus flavimaculatus**

ABCA4

**12 Sorsby fundus dystrophy, Pseudoinflammatory**

TIMP3

**13 Retinitis punctate albescens**

PRPH2, RHO

**14 Bietti crystalline corneoretinal dystrophy**

CYP4V2

**15 Retinopathy of prematurity**

FZD4

**16 Choriodal dystrophy,** **central areolar 2**

PRPH2

**17 Vitreoretino choroidopathy**

BEST1

**18 Sveinsson Chorioretinal Atrophy**

TEAD1

**19 Gyrate atrophy of choroid and retina with or without ornithinemia**

OAT

**20 Retinoschisis, X-linked**

RS1

**21 Vitreoretinopathy**

NDP, TSPAN12, FZD4, LRP5, COL2A1, KCNJ13

**22 Microphthalmi**

BCOR, SOX2, OTX2, BMP4, HCCS, STRA6, SIX6, RAX, CRYBA4,GDF6, SHH

**23 Nystagmus**

FRMD7, GPR143

**24 Optic Atrophy**

OPA1, OPA3, TMEM126A

**25 Corneal dystrophy**

TGFBI, UBIAD1, CHST6, VSX1, PIKFYVE, DCN, KRT12, KRT3

**26 Macular degeneration**

HMCN1, ABCA4, FBLN5, RAX2, CNGB3, RPGR, BEST1, GUCA1B, C1QTNF5, IMPG2, ELOVL4, PRPH2, RP1L1, CDH3, PROM1

**27 Aniridia**

PAX6
