# Supplementary material for: Next-Generation Sequencing-Aided Rapid Molecular Diagnosis of Occult Macular Dystrophy in a Chinese Family
Source: Front Genet. 2017 Aug 25;8:107. doi: 10.3389/fgene.2017.00107 (PMC5574873; doi:10.3389/fgene.2017.00107)
Supplement: TABLE S2 — The 130 eye disease-related genes included in the capture panel. [file Table_2.doc]

| **Number** | **Genes*** | **RefSeq*** | **Nucleic Acid Alteration** | **Amino Acid Alteration** | **Mutation Location** | **Zygosity *** | **Chr:Location** | **RS-ID** | **FrequencyIn1000Genomes** |
| --- | --- | --- | --- | --- | --- | --- | --- | --- | --- |
| 1 | CDHR1 | NM_033100 | c.477A>G | p.Ala159Ala | EX6 | Het | chr10:85960395 | rs4933975 | 0.4625 |
| 2 | CDHR1 | NM_033100 | c.2439T>C | p.Thr813Thr | EX17 | Hom | chr10:85974236 | rs3814213 | 0.3452 |
| 3 | RGR | NM_002921 | c.27T>C | p.Thr9Thr | EX1 | Hom | chr10:86004873 | rs2279227 | 0.4112 |
| 4 | PDE6C | NM_006204 | c.808T>A | p.Ser270Thr | EX4 | Het | chr10:95381773 | rs701865 | 0.4533 |
| 5 | PDE6C | NM_006204 | c.1098G>A | p.Ala366Ala | EX8 | Het | chr10:95389041 | rs714550 | 0.456 |
| 6 | PDE6C | NM_006204 | c.1270-7A>G | - | IN9 | Hom | chr10:95395247 | rs616522 | 0.5046 |
| 7 | PDE6C | NM_006204 | c.1935+10C>A | - | IN15 | Het | chr10:95405814 | rs1409332 | 0.4249 |
| 8 | PDE6C | NM_006204 | c.2466G>A | p.Lys822Lys | EX21 | Het | chr10:95422883 | rs79487435 | 0.0055 |
| 9 | OAT | NM_000274 | c.1134C>T | p.Asn378Asn | EX9 | Het | chr10:126089434 | rs11461 | 0.4139 |
| 10 | TEAD1 | NM_021961 | c.513C>T | p.Asp171Asp | EX8 | Hom | chr11:12903443 | rs2304733 | 0.2802 |
| 11 | BEST1 | NM_004183 | c.109T>C | p.Leu37Leu | EX2 | Hom | chr11:61719387 | rs1800007 | 0.4469 |
| 12 | BEST1 | NM_004183 | c.219C>A | p.Ile73Ile | EX3 | Hom | chr11:61722645 | rs1109748 | 0.1474 |
| 13 | BEST1 | NM_004183 | c.1608T>C | p.Thr536Thr | EX10 | Hom | chr11:61730234 | rs1800009 | 0.3553 |
| 14 | LRP5 | NM_002335 | c.1647T>C | p.Phe549Phe | EX8 | Hom | chr11:68171013 | rs545382 | 0.7665 |
| 15 | LRP5 | NM_002335 | c.2220C>T | p.Asn740Asn | EX10 | Het | chr11:68177510 | rs2306862 | 0.2262 |
| 16 | LRP5 | NM_002335 | c.3357G>A | p.Val1119Val | EX15 | Het | chr11:68192690 | rs556442 | 0.3874 |
| 17 | LRP5 | NM_002335 | c.3361A>G | p.Asn1121Asp | EX15 | Het | chr11:68192694 | rs80358317 | 0.0064 |
| 18 | TMEM126A | NM_032273 | c.395+10A>G | - | IN4 | Het | chr11:85366762 | rs2196168 | 0.4121 |
| 19 | C1QTNF5 | NM_015645 | c.-1250+3G>A | - | IN11 | Het | chr11:119213303 | rs11217241 | 0.228 |
| 20 | CACNA2D4 | NM_172364 | c.2316C>T | p.Phe772Phe | EX24 | Het | chr12:1955786 | rs11836202 | 0.2161 |
| 21 | CACNA2D4 | NM_172364 | c.2035C>T | p.Leu679Leu | EX21 | Het | chr12:1965369 | rs2286372 | 0.2244 |
| 22 | CACNA2D4 | NM_172364 | c.979A>G | p.Ile327Val | EX8 | Hom | chr12:1995403 | rs10735005 | 0.8681 |
| 23 | PDE6H | NM_006205 | c.195A>G | p.Pro65Pro | EX4 | Het | chr12:15134353 | rs2230872 | 0.2234 |
| 24 | COL2A1 | NM_001844 | c.4213G>A | p.Gly1405Ser | EX53 | Het | chr12:48367976 | rs2070739 | 0.2674 |
| 25 | KRT3 | NM_057088 | c.1089C>T | p.Arg363Arg | EX5 | Hom | chr12:53186122 | rs4432093 | 0.7106 |
| 26 | GRK1 | NM_002929 | c.700-5T>C | - | IN1 | Het | chr13:114323997 | rs9796234 | 0.4625 |
| 27 | RPGRIP1 | NM_020366 | c.3097G>C | p.Glu1033Gln | EX18 | Het | chr14:21796784 | rs3748361 | 0.3581 |
| 28 | BMP4 | NM_130850 | c.455T>C | p.Val152Ala | EX4 | Het | chr14:54417522 | rs17563 | 0.4231 |
| 29 | SIX6 | NM_007374 | c.421C>A | p.His141Asn | EX1 | Het | chr14:60976537 | rs33912345 | 0.3571 |
| 30 | SPATA7 | NM_018418 | c.220G>A | p.Val74Met | EX4 | Het | chr14:88862529 | rs3179969 | 0.4505 |
| 31 | FBLN5 | NM_006329 | c.945T>C | p.Ile315Ile | EX9 | Het | chr14:92347680 | rs2430347 | 0.3159 |
| 32 | TRPM1 | NM_002420 | c.4494T>A | p.His1498Gln | EX27 | Het | chr15:31294343 | rs12898290 | 0.0989 |
| 33 | TRPM1 | NM_002420 | c.4123G>T | p.Glu1375Ter | EX27 | Het | chr15:31294714 | rs3784589 | 0.0989 |
| 34 | TRPM1 | NM_002420 | c.2340T>C | p.Asn780Asn | EX18 | Hom | chr15:31330280 | rs2288242 | 0.6749 |
| 35 | TRPM1 | NM_002420 | c.1239G>A | p.Thr413Thr | EX11 | Hom | chr15:31342744 | rs1035705 | 0.391 |
| 36 | TRPM1 | NM_002420 | c.95G>A | p.Ser32Asn | EX3 | Hom | chr15:31362352 | rs2241493 | 0.6969 |
| 37 | TRPM1 | NM_002420 | c.2T>C | p.Met1Thr | EX2 | Hom | chr15:31369123 | rs4779816 | 0.7793 |
| 38 | SLC24A1 | NM_004727 | c.109A>T | p.Thr37Ser | EX2 | Het | chr15:65916527 | rs3743171 | 0.3114 |
| 39 | SLC24A1 | NM_004727 | c.175C>G | p.Pro59Ala | EX2 | Het | chr15:65916593 | rs533092441 | 0 |
| 40 | STRA6 | NM_001199040 | c.1278-10C>G | - | IN13 | Het | chr15:74476340 | rs2277608 | 0.2976 |
| 41 | CNGB1 | NM_001297 | c.2732A>G | p.Lys911Arg | EX27 | Het | chr16:57937788 | rs2303785 | 0.1603 |
| 42 | CNGB1 | NM_001297 | c.2664C>G | p.Ala888Ala | EX27 | Het | chr16:57937856 | rs413562 | 0 |
| 43 | CNGB1 | NM_001297 | c.2635-10C>T | - | IN26 | Het | chr16:57937895 | rs437920 | 0.3562 |
| 44 | CNGB1 | NM_001297 | c.2193C>T | p.Asn731Asn | EX22 | Hom | chr16:57950057 | rs376270 | 0.1328 |
| 45 | CNGB1 | NM_001297 | c.327C>T | p.Gly109Gly | EX5 | Het | chr16:57996932 | rs17821448 | 0.4002 |
| 46 | CNGB1 | NM_001297 | c.299G>A | p.Arg100His | EX5 | Het | chr16:57996960 | rs13336595 | 0.348 |
| 47 | AIPL1 | NM_014336 | c.651A>G | p.Pro217Pro | EX5 | Het | chr17:6330068 | rs2292546 | 0.4203 |
| 48 | PITPNM3 | NM_031220 | c.2430T>C | p.Asp810Asp | EX18 | Het | chr17:6364753 | rs11654099 | 0.1694 |
| 49 | GUCY2D | NM_000180 | c.741C>T | p.His247His | EX3 | Het | chr17:7907189 | rs3829789 | 0.2253 |
| 50 | GUCY2D | NM_000180 | c.2101C>T | p.Pro701Ser | EX10 | Het | chr17:7915912 | rs34598902 | 0.1355 |
| 51 | KRT12 | NM_000223 | c.43C>T | p.Pro15Ser | EX1 | Het | chr17:39023396 | rs11650915 | 0.38 |
| 52 | FSCN2 | NM_012418 | c.732G>A | p.Thr244Thr | EX1 | Het | chr17:79496289 | rs34797307 | 0.0559 |
| 53 | PRPF31 | NM_015629 | c.138T>C | p.Asp46Asp | EX2 | Het | chr19:54621796 | rs76251057 | 0.0046 |
| 54 | PRPF31 | NM_015629 | c.1147-9T>C | - | IN11 | Hom | chr19:54632423 | rs655240 | 0.5943 |
| 55 | DHDDS | NM_024887 | c.757G>A | p.Val253Met | EX8 | Het | chr1:26786627 | rs3816539 | 0.4249 |
| 56 | RPE65 | NM_000329 | c.1056G>A | p.Glu352Glu | EX10 | Het | chr1:68903942 | rs12145904 | 0.3361 |
| 57 | ABCA4 | NM_000350 | c.6119G>A | p.Arg2040Gln | EX44 | Het | chr1:94471025 | rs148460146 | 0.0009 |
| 58 | ABCA4 | NM_000350 | c.6069T>C | p.Ile2023Ile | EX44 | Hom | chr1:94471075 | rs1762114 | 0.6905 |
| 59 | GNAT2 | NM_005272 | c.546G>A | p.Thr182Thr | EX5 | Hom | chr1:110148974 | rs1799875 | 0.2372 |
| 60 | HMCN1 | NM_031935 | c.4857T>C | p.His1619His | EX31 | Het | chr1:185984517 | rs6665753 | 0.4057 |
| 61 | HMCN1 | NM_031935 | c.7253T>C | p.Ile2418Thr | EX46 | Het | chr1:186026474 | rs12129650 | 0.4927 |
| 62 | HMCN1 | NM_031935 | c.7371C>T | p.Cys2457Cys | EX47 | Het | chr1:186031041 | rs7522627 | 0.4505 |
| 63 | HMCN1 | NM_031935 | c.8678A>G | p.Glu2893Gly | EX56 | Het | chr1:186050417 | rs10798035 | 0.4634 |
| 64 | HMCN1 | NM_031935 | c.8885-7A>G | - | IN57 | Het | chr1:186055371 | rs6425017 | 0.4112 |
| 65 | HMCN1 | NM_031935 | c.12230-7A>C | - | IN80 | Het | chr1:186092076 | rs2057388 | 0.4038 |
| 66 | HMCN1 | NM_031935 | c.13310A>G | p.Gln4437Arg | EX86 | Het | chr1:186101539 | rs10911825 | 0.4377 |
| 67 | FLVCR1 | NM_014053 | c.154G>C | p.Ala52Pro | EX1 | Het | chr1:213031948 | rs11120047 | 0.4615 |
| 68 | FLVCR1 | NM_014053 | c.1593+9T>C | - | IN9 | Het | chr1:213068404 | rs17019870 | 0.369 |
| 69 | FLVCR1 | NM_014053 | c.1631C>T | p.Thr544Met | EX10 | Het | chr1:213068595 | rs3207090 | 0.4396 |
| 70 | USH2A | NM_206933 | c.13191G>A | p.Glu4397Glu | EX63 | Hom | chr1:215848062 | rs2009923 | 0.0449 |
| 71 | USH2A | NM_206933 | c.10232A>C | p.Glu3411Ala | EX52 | Het | chr1:215960167 | rs10864198 | 0.4808 |
| 72 | USH2A | NM_206933 | c.6506T>C | p.Ile2169Thr | EX34 | Het | chr1:216172380 | rs10864219 | 0.4341 |
| 73 | USH2A | NM_206933 | c.3812-8T>G | - | IN17 | Het | chr1:216371934 | rs646094 | 0.3159 |
| 74 | USH2A | NM_206933 | c.504A>G | p.Thr168Thr | EX3 | Hom | chr1:216592003 | rs4253963 | 0.4386 |
| 75 | USH2A | NM_206933 | c.373G>A | p.Ala125Thr | EX2 | Hom | chr1:216595306 | rs10779261 | 0.5806 |
| 76 | IDH3B | NM_174855 | c.118-3G>C | - | IN2 | Hom | chr20:2644407 | rs2073193 | 0.4121 |
| 77 | CRYBA4 | NM_001886 | c.171T>C | p.Phe57Phe | EX4 | Hom | chr22:27021457 | rs5761637 | 0.848 |
| 78 | TIMP3 | NM_000362 | c.249T>C | p.His83His | EX3 | Het | chr22:33253280 | rs9862 | 0.4194 |
| 79 | TIMP3 | NM_000362 | c.261C>T | p.Ser87Ser | EX3 | Het | chr22:33253292 | rs11547635 | 0.391 |
| 80 | FAM161A | NM_032180 | c.1212T>C | p.Cys404Cys | EX3 | Hom | chr2:62066927 | rs4672457 | 0.9579 |
| 81 | FAM161A | NM_032180 | c.321A>G | p.Ile107Met | EX2 | Het | chr2:62069358 | rs11125895 | 0.2454 |
| 82 | SNRNP200 | NM_014014 | c.5317C>T | p.Leu1773Leu | EX37 | Het | chr2:96944553 | rs772175 | 0.3764 |
| 83 | SNRNP200 | NM_014014 | c.5134-6C>G | - | IN36 | Het | chr2:96944742 | rs3214062 | 0.0055 |
| 84 | SNRNP200 | NM_014014 | c.3654C>T | p.Ser1218Ser | EX28 | Het | chr2:96952601 | rs3214060 | 0.0412 |
| 85 | MERTK | NM_006343 | c.353G>A | p.Ser118Asn | EX2 | Het | chr2:112686988 | rs13027171 | 0.2527 |
| 86 | MERTK | NM_006343 | c.583+10C>T | - | IN3 | Het | chr2:112702647 | rs11683694 | 0.2857 |
| 87 | MERTK | NM_006343 | c.1397G>A | p.Arg466Lys | EX9 | Het | chr2:112751928 | rs7604639 | 0.4066 |
| 88 | MERTK | NM_006343 | c.1494C>T | p.Asn498Asn | EX10 | Het | chr2:112754943 | rs3811634 | 0.2747 |
| 89 | MERTK | NM_006343 | c.1552A>G | p.Ile518Val | EX10 | Het | chr2:112755001 | rs2230515 | 0.4057 |
| 90 | MERTK | NM_006343 | c.1881A>G | p.Ser627Ser | EX14 | Het | chr2:112765973 | rs1131244 | 0.4112 |
| 91 | CERKL | NM_001160277 | c.1452C>T | p.Asp484Asp | EX12 | Hom | chr2:182403851 | rs10180793 | 0.9799 |
| 92 | CERKL | NM_001160277 | c.242A>C | p.Asp81Ala | EX2 | Het | chr2:182468803 | rs61750041 | 0.3004 |
| 93 | CERKL | NM_001160277 | c.156C>T | p.Phe52Phe | EX1 | Het | chr2:182521578 | rs1473295 | 0.4496 |
| 94 | PIKFYVE | NM_015040 | c.2087G>A | p.Ser696Asn | EX17 | Hom | chr2:209184980 | rs10932258 | 0.9863 |
| 95 | PIKFYVE | NM_015040 | c.2106C>T | p.Pro702Pro | EX17 | Hom | chr2:209184999 | rs10932259 | 0.881 |
| 96 | PIKFYVE | NM_015040 | c.2795T>C | p.Leu932Ser | EX20 | Hom | chr2:209190330 | rs2363468 | 0.8819 |
| 97 | PIKFYVE | NM_015040 | c.2984A>T | p.Gln995Leu | EX20 | Hom | chr2:209190519 | rs893254 | 0.8388 |
| 98 | PIKFYVE | NM_015040 | c.2993C>G | p.Thr998Ser | EX20 | Hom | chr2:209190528 | rs893253 | 0.8791 |
| 99 | PIKFYVE | NM_015040 | c.3547C>A | p.Gln1183Lys | EX20 | Hom | chr2:209191082 | rs1529979 | 0.881 |
| 100 | PIKFYVE | NM_015040 | c.3564T>C | p.Asn1188Asn | EX20 | Hom | chr2:209191099 | rs1529978 | 0.9908 |
| 101 | PIKFYVE | NM_015040 | c.5028-8G>A | - | IN32 | Hom | chr2:209209827 | rs2304544 | 0.88 |
| 102 | PIKFYVE | NM_015040 | c.5334G>A | p.Thr1778Thr | EX35 | Hom | chr2:209212707 | rs2304545 | 0.6291 |
| 103 | PIKFYVE | NM_015040 | c.5397A>G | p.Thr1799Thr | EX36 | Hom | chr2:209214770 | rs2118297 | 0.881 |
| 104 | PIKFYVE | NM_015040 | c.5526A>G | p.Glu1842Glu | EX37 | Hom | chr2:209215586 | rs994697 | 0.881 |
| 105 | SAG | NM_000541 | c.1207G>A | p.Val403Ile | EX16 | Het | chr2:234255547 | rs1046974 | 0.3947 |
| 106 | IMPG2 | NM_016247 | c.3381C>T | p.Leu1127Leu | EX16 | Hom | chr3:100949842 | rs348867 | 0.6767 |
| 107 | IMPG2 | NM_016247 | c.2021C>T | p.Thr674Ile | EX13 | Het | chr3:100963154 | rs571391 | 0.4185 |
| 108 | IMPG2 | NM_016247 | c.666+10G>A | - | IN6 | Het | chr3:100994497 | rs533852 | 0.4295 |
| 109 | OPA1 | NM_015560 | c.870+4T>C | - | IN8 | Hom | chr3:193355074 | rs166850 | 0.8306 |
| 110 | OPA1 | NM_015560 | c.2808G>A | p.Ala936Ala | EX27 | Het | chr3:193385059 | rs117475774 | 0.0586 |
| 111 | CYP4V2 | NM_207352 | c.846T>C | p.Cys282Cys | EX7 | Hom | chr4:187122355 | rs3736456 | 0.0394 |
| 112 | CYP4V2 | NM_207352 | c.1507G>C | p.Gly503Arg | EX11 | Het | chr4:187131724 | - | 0 |
| 113 | TGFBI | NM_000358 | c.651G>C | p.Leu217Leu | EX6 | Het | chr5:135382989 | rs1442 | 0.3947 |
| 114 | GRM6 | NM_000843 | c.2196G>A | p.Thr732Thr | EX9 | Het | chr5:178410151 | rs2071247 | 0.3645 |
| 115 | GRM6 | NM_000843 | c.1392A>G | p.Gly464Gly | EX7 | Hom | chr5:178413947 | rs11746675 | 0.3379 |
| 116 | GRM6 | NM_000843 | c.1308T>C | p.Thr436Thr | EX6 | Hom | chr5:178415982 | rs4701014 | 0.9771 |
| 117 | GRM6 | NM_000843 | c.1227C>T | p.Tyr409Tyr | EX6 | Hom | chr5:178416063 | rs2645339 | 0.2766 |
| 118 | GRM6 | NM_000843 | c.1131C>T | p.Asp377Asp | EX5 | Hom | chr5:178416288 | rs2071246 | 0.5394 |
| 119 | MAK | NM_005906 | c.1830T>C | p.His610His | EX13 | Hom | chr6:10764727 | rs126405 | 0.7546 |
| 120 | TULP1 | NM_003322 | c.783G>C | p.Lys261Asn | EX8 | Hom | chr6:35477025 | rs2064318 | 0.7326 |
| 121 | TULP1 | NM_003322 | c.776T>C | p.Ile259Thr | EX8 | Het | chr6:35477032 | rs2064317 | 0.4808 |
| 122 | TULP1 | NM_003322 | c.200C>G | p.Thr67Arg | EX4 | Hom | chr6:35479574 | rs7764472 | 0.7894 |
| 123 | GUCA1B | NM_002098 | c.171T>C | p.Tyr57Tyr | EX1 | Hom | chr6:42162388 | rs3749921 | 0.4212 |
| 124 | PRPH2 | NM_000322 | c.1013A>G | p.Asp338Gly | EX3 | Het | chr6:42666061 | rs434102 | 0.3452 |
| 125 | PRPH2 | NM_000322 | c.929G>A | p.Arg310Lys | EX3 | Hom | chr6:42666145 | rs425876 | 0.8929 |
| 126 | PRPH2 | NM_000322 | c.910C>G | p.Gln304Glu | EX3 | Het | chr6:42666164 | rs390659 | 0.3471 |
| 127 | PRPH2 | NM_000322 | c.318T>C | p.Val106Val | EX1 | Het | chr6:42689755 | rs7764439 | 0.4799 |
| 128 | EYS | NM_001142800 | c.7666A>T | p.Ser2556Cys | EX39 | Hom | chr6:64498055 | rs66462731 | 0.0174 |
| 129 | EYS | NM_001142800 | c.6977G>A | p.Arg2326Gln | EX35 | Hom | chr6:64694354 | rs4710457 | 0.1209 |
| 130 | EYS | NM_001142800 | c.5705A>T | p.Asn1902Ile | EX27 | Hom | chr6:65149185 | rs9353806 | 0.1053 |
| 131 | EYS | NM_001142800 | c.4256T>C | p.Leu1419Ser | EX26 | Hom | chr6:65301504 | rs624851 | 0.5723 |
| 132 | EYS | NM_001142800 | c.3444-5C>T | - | IN22 | Het | chr6:65336143 | rs9445051 | 0.3168 |
| 133 | EYS | NM_001142800 | c.2555T>C | p.Leu852Pro | EX16 | Het | chr6:65622463 | rs9294631 | 0.4057 |
| 134 | EYS | NM_001142800 | c.1891G>A | p.Gly631Ser | EX12 | Hom | chr6:66005888 | rs9342464 | 0.446 |
| 135 | EYS | NM_001142800 | c.1809C>T | p.Val603Val | EX12 | Hom | chr6:66005970 | rs9345601 | 0.446 |
| 136 | RIMS1 | NM_014989 | c.666A>G | p.Leu222Leu | EX5 | Hom | chr6:72889472 | rs2249021 | 0.5678 |
| 137 | RIMS1 | NM_014989 | c.743C>T | p.Ser248Leu | EX5 | Het | chr6:72889549 | rs116476753 | 0.0339 |
| 138 | RIMS1 | NM_014989 | c.1083A>G | p.Leu361Leu | EX6 | Hom | chr6:72892257 | rs2463730 | 0.9707 |
| 139 | RIMS1 | NM_014989 | c.1938A>C | p.Val646Val | EX9 | Het | chr6:72947606 | - | 0 |
| 140 | LCA5 | NM_001122769 | c.71T>C | p.Leu24Ser | EX2 | Hom | chr6:80228541 | rs2655655 | 0.7958 |
| 141 | RP9 | NM_203288 | c.629A>G | p.Lys210Arg | EX6 | Het | chr7:33134883 | rs150987618 | 0.2647 |
| 142 | RP9 | NM_203288 | c.314-9C>T | - | IN3 | Hom | chr7:33136983 | rs6462460 | 0.989 |
| 143 | TSPAN12 | NM_012338 | c.765G>T | p.Pro255Pro | EX8 | Het | chr7:120428799 | rs41623 | 0.2775 |
| 144 | RP1L1 | NM_178857 | c.6853G>A | p.Gly2285Arg | EX4 | Hom | chr8:10464755 | rs55642448 | 0.2189 |
| 145 | RP1L1 | NM_178857 | c.6723A>G | p.Ser2241Ser | EX4 | Hom | chr8:10464885 | rs56382513 | 0.4725 |
| 146 | RP1L1 | NM_178857 | c.5860A>G | p.Thr1954Ala | EX4 | Hom | chr8:10465748 | rs11783478 | 0.4679 |
| 147 | RP1L1 | NM_178857 | c.5837C>A | p.Ala1946Glu | EX4 | Hom | chr8:10465771 | rs11785822 | 0.8901 |
| 148 | RP1L1 | NM_178857 | c.4448C>T | p.Ala1483Val | EX4 | Hom | chr8:10467160 | rs62490855 | 0.0247 |
| 149 | RP1L1 | NM_178857 | c.4401G>T | p.Arg1467Ser | EX4 | Hom | chr8:10467207 | rs4840498 | 0.9185 |
| 150 | RP1L1 | NM_178857 | c.4019A>G | p.Glu1340Gly | EX4 | Hom | chr8:10467589 | rs9657518 | 0 |
| 151 | RP1L1 | NM_178857 | c.4004G>T | p.Gly1335Val | EX4 | Hom | chr8:10467604 | rs74366179 | 0.0311 |
| 152 | RP1L1 | NM_178857 | c.3436C>T | p.Arg1146Trp | EX4 | Hom | chr8:10468172 | rs4840502 | 0.4661 |
| 153 | RP1L1 | NM_178857 | c.2578C>T | p.Arg860Trp | EX4 | Hom | chr8:10469030 | rs62490856 | 0.0247 |
| 154 | RP1L1 | NM_178857 | c.2268C>T | p.Asn756Asn | EX4 | Hom | chr8:10469340 | rs57819090 | 0.0247 |
| 155 | RP1L1 | NM_178857 | c.1791C>T | p.Gly597Gly | EX4 | Hom | chr8:10469817 | rs6996950 | 0.6163 |
| 156 | RP1L1 | NM_178857 | c.501A>G | p.Thr167Thr | EX2 | Het | chr8:10480211 | rs79329877 | 0.0476 |
| 157 | RP1L1 | NM_178857 | c.133C>T | p.Arg45Trp | EX2 | Het | chr8:10480579 | rs267607017 | 0 |
| 158 | CNGB3 | NM_019098 | c.2264A>G | p.Glu755Gly | EX18 | Het | chr8:87588198 | rs3735972 | 0.1474 |
| 159 | CNGB3 | NM_019098 | c.2214A>G | p.Glu738Glu | EX18 | Het | chr8:87588248 | rs3735970 | 0.1474 |
| 160 | CNGB3 | NM_019098 | c.892A>C | p.Thr298Pro | EX7 | Het | chr8:87666251 | rs4961206 | 0.4478 |
| 161 | CNGB3 | NM_019098 | c.702T>G | p.Cys234Trp | EX6 | Hom | chr8:87679303 | rs6471482 | 0.8709 |
| 162 | KCNV2 | NM_133497 | c.183C>G | p.Gly61Gly | EX1 | Hom | chr9:2717922 | rs10967705 | 0.4341 |
| 163 | BCOR | NM_001123384 | c.1692A>G | p.Ala564Ala | EX4 | Hemi | chrX:39932907 | rs6520618 | 0.1575 |
| 164 | BCOR | NM_001123384 | c.1260T>C | p.Asp420Asp | EX4 | Hemi | chrX:39933339 | rs5917933 | 0.4524 |
| 165 | CACNA1F | NM_005183 | c.3309T>C | p.His1103His | EX28 | Hemi | chrX:49071964 | rs2075866 | 0.1813 |
| 166 | CACNA1F | NM_005183 | c.1842C>T | p.Gly614Gly | EX14 | Hemi | chrX:49081291 | rs2235127 | 0.1227 |
| 167 | CACNA1F | NM_005183 | c.1118+6G>A | - | IN8 | Hemi | chrX:49084492 | rs6609854 | 0.0916 |
| 168 | KRT3 | NM_057088 | c.396_413 delAGGGGCTGGTGGCTTTGG | - | - | Hom | chr12:53189414..53189431 | rs148531142 | 0 |
| 169 | CEP290 | NM_025114 | c.3574 -9 delT | - | - | Hom | chr12:88483273 | rs10717563 | 0 |
